# Multimodal Brain Connectomics-Based Prediction of Parkinson’s Disease Using Graph Attention Networks

**DOI:** 10.3389/fnins.2021.741489

**Published:** 2022-02-23

**Authors:** Apoorva Safai, Nirvi Vakharia, Shweta Prasad, Jitender Saini, Apurva Shah, Abhishek Lenka, Pramod Kumar Pal, Madhura Ingalhalikar

**Affiliations:** ^1^Symbiosis Center for Medical Image Analysis, Symbiosis Institute of Technology, Symbiosis International University, Pune, India; ^2^Department of Neurology, National Institute of Mental Health and Neurosciences, Bengaluru, India; ^3^Department of Clinical Neuroscience, National Institute of Mental Health and Neurosciences, Bengaluru, India; ^4^Department of Neuroimaging and Interventional Radiology, National Institute of Mental Health and Neurosciences, Bengaluru, India

**Keywords:** multimodal brain connectivity, structure-function, Parkinson’s disease, graph attention network, interpretability

## Abstract

**Background:**

A multimodal connectomic analysis using diffusion and functional MRI can provide complementary information on the structure–function network dynamics involved in complex neurodegenerative network disorders such as Parkinson’s disease (PD). Deep learning-based graph neural network models generate higher-level embeddings that could capture intricate structural and functional regional interactions related to PD.

**Objective:**

This study aimed at investigating the role of structure–function connections in predicting PD, by employing an end-to-end graph attention network (GAT) on multimodal brain connectomes along with an interpretability framework.

**Methods:**

The proposed GAT model was implemented to generate node embeddings from the structural connectivity matrix and multimodal feature set containing morphological features and structural and functional network features of PD patients and healthy controls. Graph classification was performed by extracting topmost node embeddings, and the interpretability framework was implemented using saliency analysis and attention maps. Moreover, we also compared our model with unimodal models as well as other state-of-the-art models.

**Results:**

Our proposed GAT model with a multimodal feature set demonstrated superior classification performance over a unimodal feature set. Our model demonstrated superior classification performance over other comparative models, with 10-fold CV accuracy and an F1 score of 86% and a moderate test accuracy of 73%. The interpretability framework highlighted the structural and functional topological influence of motor network and cortico-subcortical brain regions, among which structural features were correlated with onset of PD. The attention maps showed dependency between large-scale brain regions based on their structural and functional characteristics.

**Conclusion:**

Multimodal brain connectomic markers and GAT architecture can facilitate robust prediction of PD pathology and provide an attention mechanism-based interpretability framework that can highlight the pathology-specific relation between brain regions.

## Introduction

Brain connectomics is an emerging whole-brain network approach to neuroscience that is based on magnetic resonance imaging (MRI) of the brain. In comparison to traditional neuroimaging analysis techniques such as regional or voxel-wise analysis, connectomics can capture a higher-order interaction or relation between various brain regions. Over the years, connectome-based delineation of neuropathology has been performed using different MRI modalities such as diffusion-weighted imaging (DWI) and functional MRI (fMRI) (Hayes et al., 2016; Tessitore et al., 2016; Hohenfeld et al., 2018). Functional connectivity (FC) derived from functional MRI reflects time-dependent neuronal synchronization between brain regions anchored through an anatomical infrastructure of white matter pathways called structural connectivity (SC) which is computed from diffusion MRI. The interrelation between the structure–function topography is of high interest in disentangling the complexities of the large-scale dynamics of the brain. Moreover, the deviation of structure–function coupling in neurodegenerative pathology such as Parkinson’s disease is intriguing. Novel graph-based deep learning techniques could be one possible means to unravel complex structure–function dynamics and facilitate deeper insights into aberrant structural pathways and associated functional disruptions in several neurodegenerative disorders.

The brain connectomes are essentially graph matrices, where nodes represent the brain regions and edges indicate the physical (structural) or functional link between different regions. Owing to the non-Euclidean, graphical nature of the connectome, it is crucial to employ sophisticated graph embedding techniques that can represent the graphs in a lower-dimensional space, while preserving its structure and relation between nodes (Cai et al., 2018). Classical graph embedding techniques such as dimensionality reduction and matrix factorization methods, although easy to implement, have certain drawbacks; for example, they cannot represent higher-order proximities of a graph, have high time complexity, and are deterministic rather than learnable (Chen et al., 2020). Random walks-based embedding techniques for brain connectivity depend on a randomized sampling strategy and do not consider node features while generating embeddings, whereas neural network-based machine learning algorithms use hand-engineered connectivity or network measures (Chen et al., 2020). To this end, graph neural networks (GNNs) are powerful deep learning-based node embedding models that can overcome these limitations, by extracting meaningful topological features and interaction patterns from the graph, in an end-to-end learnable framework. GNNs are designed to generate embeddings for a node by aggregating features of its neighboring nodes, for either a node classification, graph classification, or link prediction task (Zhou et al., 2020). In recent years, neuroimaging studies have employed task-specific variants of graph convolution networks (GCNs), which is a popular GNN model that generalizes the convolution neural network (CNN) architecture on graph-structured data (Parisot et al., 2018; Zhang et al., 2018; Jansson and Sandström, 2020; Jiang et al., 2020; Li X. et al., 2020; Goli, 2021; Liu et al., 2021; Qu et al., 2021; Wang et al., 2021; Yao et al., 2021). The graph attention network (GAT) is another powerful GNN model which generates node embeddings by employing a self-attention mechanism, where certain nodes in the neighborhood are given more attention over others, thereby focusing on the most relevant part of the graph (Veličković et al., 2017). The key properties of GAT that make it more suitable over other GNN models for a neuroimaging-based application are that it can deal with variable-sized input features, it can perform multiple attention mechanisms parallelly that capture different aspects or projections of the data, and it can be employed in an inductive learning task, whereby the model can be generalized to perform accurately on unseen graphs (Veličković et al., 2017).

Neuroimaging studies have developed and employed machine learning frameworks for performing a brain connectome-based multimodal classification of the diseased population by fusing brain connectivity with a phenotypic score, a clinical variable, or a genetic marker (Ingalhalikar et al., 2012; Calhoun and Sui, 2016; Bi et al., 2019; Markello et al., 2021). Recently, neuroimaging studies have begun exploring GNNs on multimodal brain connectomes by using structural and FC features together or in combination with a phenotypic score. A GCN-based encoder–decoder model was developed by Li Y. et al. (2020) to classify drinkers and non-drinkers by simultaneously reconstructing FC from SC networks and extracting relationships between them, yielding a 74% classification accuracy. Another study predicted early mild cognitive impairment from the Alzheimer’s Disease Neuroimaging Initiative (ADNI) dataset by implementing GCN on a population graph, constructed using phenotypic and multimodal imaging (Liu et al., 2020). Joint structural and functional MRI analyses were performed using GCN where relational information between nodes was attained from T1w structural measures and functional brain summaries were obtained using fMRI for classifying autistic patients (Arya et al., 2020). More recently, a multimodal GCN (M-GCN) framework was proposed to predict phenotypic measures by considering the FC as input, guided by subject-wise structural connectomes (Dsouza et al., 2021). Application of the GAT model and its potential interpretability framework was first demonstrated on a bipolar dataset using the FC matrix as the graph and feature set containing anatomical and statistical FC features (Yang et al., 2019). This study implemented a dense hierarchical pooling strategy with a biological motivation that brain networks contain a community structure. However, this may introduce a prior bias and its effectiveness across different pathologies needs to be tested. Although the incorporation of edge features in GAT could enhance model performance, the inclusion of a dense FC matrix may, however, include spurious connections which may adversely affect the attention mechanism by generating irrelevant attention heads and thus ultimately affecting the model training. Nevertheless, this study encourages the utility of GAT architecture for a detailed modality-specific interpretation on model predictions.

Deep models that can employ and interpret multimodal connectomic data have not yet been introduced and applied to understand complex neurodegenerative disorders such as PD, which includes alterations in the motor network as well as variable changes in multiple brain subnetworks, which could be associated with non-motor symptoms like cognitive impairment, freezing of gait, hallucinations, mood disturbance, sleep disorders, and olfactory dysfunction (Tessitore et al., 2016, 2019). In this work, we propose a novel GAT architecture for graph classification, using multimodal brain connectomics that can accurately predict PD and additionally provides a comprehensive interpretability framework highlighting the intricate structure–function interactive patterns related to the pathology of PD. Our GAT model is employed on SC, FC, and morphological features such as cortical thickness and is anchored to SC for computing node embeddings. The most discriminative node embeddings are fetched using a top-k approach which substantially reduces the dimensionality of the feature set for the final classification task. Moreover, we provide an interpretability framework using saliency analysis that highlights the influence of structural and functional nodal features and attention maps that portrays the relation between brain regions, facilitating a deeper insight into model predictions and its clinical interpretability.

## Materials and Methods

### Data Acquisition and Processing

The dataset for this study consists of multiple MRI sequences of 75 patients with PD (age = 57.70 ± 7.41, M/F = 65/10) and 34 healthy controls (age = 56.05 ± 5.27, M/F = 26/8), scanned on a Philips 3T MRI. T1-weighted images were acquired using TR/TE 8.06/3.6 ms, voxel size 1 × 1 × 1 mm, and flip angle = 8; functional MRI (fMRI) was acquired using TR/TE 2,000/35 ms, voxel size 1.65 × 1.65 × 3 mm3, matrix size = 144 × 144, flip angle = 90, and no. of volumes 140 for 4 min and 60 s; and diffusion-weighted images (DWIs) were acquired using a single-shot spin-echo echo planar imaging (EPI) sequence with TR/TE = 8,583–9,070/60–62 ms and voxel size = 1.75 × 1.75 × 2 mm. The diffusion gradient was applied in 15 directions, with *b* value = 1,000 s/mm and a single *b* = 0 s/mm. All subjects were recruited from the general neurology outpatient clinics and Parkinson’s disease and movement disorders subspeciality clinic at the National Institute of Mental Health and Neurosciences (NIMHANS), Bangalore, India. PD was diagnosed as per the UK Parkinson’s Disease Society Brain Bank criteria. PD was diagnosed as per the UK Parkinson’s Disease Society Brain Bank criteria (Hughes et al., 1992). All subjects were screened for cognitive impairment using Mini Mental State Examination (MMSE), and a score below 24 was set as the exclusion criteria. Clinical information of patients such as disease severity and stage of disease was evaluated using the Unified Parkinson’s Disease Rating Scale (UPDRS-III) score and the modified Hoehn and Yahr (H&Y) staging system, respectively, along with variables such as age at onset (AAO) of PD, duration of illness (DOI), and Levodopa equivalent daily dosage (LEDD). Participants with any history of neuropsychiatric disorders were excluded from the study. This study was approved by the Institute Ethics Committee of NIMHANS, and informed consent was taken from all participated that were recruited for this study. The detailed demographic information of all subjects is mentioned in Table 1.

**TABLE 1:** Demographic and clinical characteristics of patients with Parkinson’s disease and healthy controls.

	Parkinson’s disease (*n* = 75)	Healthy controls (*n* = 34)
Gender (F:M)	10:65	8:26
Age (years)	57.70 ± 7.41	56.05 ± 5.27
Age at onset (years)	51.14 ± 9.08	–
Duration of illness (years)	5.87 ± 2.74	–
MMSE score	28.04 ± 1.61	
UPDRS III (OFF)	34.53 ± 8.25	
Hoehn and Yahr stage	2.31 ± 0.28	
LEDD	616.01 ± 277.65	–

*F, female; M, male; MMSE, Mini-Mental Status Examination; UPDRS, Unified Parkinson’s Disease Rating Scale; LEDD, levodopa equivalent daily dose.*

### Construction of Multimodal Brain Connectomes and Feature Set

#### T1-Image Preprocessing

The T1w images were preprocessed and parcellated into 86 brain regions of interest (ROIs), containing 68 cortical and 18 subcortical regions of the Desikan atlas (Desikan et al., 2006) using FreeSurfer (Fischl, 2012). These 86 ROIs act as nodes of the brain connectome. Preprocessing of T1w images includes skull stripping, bias correction, and tissue segmentation, followed by a surface-based non-linear registration to map cortical sulci and gyri and a volume-based registration to map the subcortical regions using FreeSurfer. Segmentation of the 86 regions was manually checked for each subject. Seed voxels in the 86 regions were located by dilating the white matter (WM) masks and intersecting it with segmented node labels to compute the gray and white matter boundary.

#### Structural Connectivity

DWI images were initially preprocessed by performing eddy current and motion correction and brain extraction. The T1-weighted images were registered to the diffusion images by employing an affine transformation, and subsequently the 86 ROIs were transferred to the diffusion space using nearest-neighbor interpolation. Structural connectomes were computed using a probabilistic tractography algorithm which performs fiber tracking by repeatedly sampling diffusion distributions at each seed voxel in the brain. Streamlines are constructed such that each sample follows the next and a probability-weighted distribution of possible fiber tracks is obtained (Behrens et al., 2007). Tracking parameters were set to 2 fibers per voxel and 5,000 sample streamlines per voxel. The connectivity measure used was conditional probability *Pij* between the seed ROI, *i*, and the target ROI, that quantifies connectivity such that *Pij*≈ *Pji*. The undirected weighted SC matrix was thresholded at 0.1 edge value which sparsened the matrix by 50%, keeping the stronger connections.

#### Functional Connectivity

Preprocessing of fMRI data included de-spiking, realignment of all functional volumes for motion correction, and co-registration of fMRI images to FreeSurfer preprocessed T1 images from Section “T1-Image Preprocessing.” The data were denoised by regressing out 6 motion parameters and the mean signal from the WM and cerebrospinal fluid (CSF) regions to reduce motion and physiological artifacts. Denoised data were band-pass filtered with a frequency range 0.01–0.1 Hz and smoothened using a kernel of 5 mm full width half maximum (FWHM). Subjects with large motion were discarded by applying a cutoff of more than 30% data having mean frame-wise displacement > 0.5 mm. FC was computed in native space using 86 ROIs from the Desikan Killiany atlas by calculating pairwise Pearson’s correlation between a mean time series of each ROI with every other ROI of the atlas. The correlation matrices were normalized using Fisher’s r to z transform and thresholded with a density cutoff of 0.5, to sparsen the matrix by eliminating the weak and retaining the strongest connections.

### Multimodal Feature Set

The multimodal feature set consists of morphological features from T1-weighted images and structural and functional features that were computed from SC and FC matrices. Morphological features indicating the structural regional attributes such as volume and cortical thickness were extracted from preprocessed T1 images using FreeSurfer software (Fischl, 2012). Network-based nodal features indicating the local topological attributes of a node such as clustering coefficient (CC), betweenness centrality (BC), degree (D), strength of connectivity (S), local efficiency (LE), modularity (Mod), and participation coefficient (PC) were computed using Brain Connectivity Toolbox (Rubinov and Sporns, 2010). Details of these graph theory measures are provided in Supplementary Material. We also computed 4 statistical features such as mean, standard deviation (std), skewness (skew), and kurtosis (kurt) on the all-edge values belonging to a particular node in the graph, indicating the overall distribution of connections, for each node. Thus, the multimodal feature set contained 24 features comprising 13 structural features (2 morphological features + 7 network + 4 statistical) and 11 functional features (7 network + 4 statistical) computed from T1-weighted images, SC and FC matrices, respectively.

### Multimodal Graph Classification Using Graph Attention Network

#### Graph Attention Network

A GAT architecture is built by stacking single attentional layers on graph-structured data (Veličković et al., 2017). The input to the GAT layer is [G(nxn), h] where G indicates the graph matrix with n nodes and h is a set of node features, $h=\left\{{\vec{h}}_{1,}{\vec{h}}_{2,},..,{\vec{h}}_{n,}\right\}$, *h*_*i*_ ∈ *R^F^*, containing F no. of features. The input features are transformed to a set of higher-level features $h^{\prime }=\left\{{\vec{h}}_{1}^{\prime },{\vec{h}}_{2}^{\prime },..,{\vec{h}}_{n}^{\prime }\right\}$, $h_{i}^{\prime }\in R^{F^{\prime }}\,w\,i\,t\,h$ cardinality *F*′. In order to obtain the new features, a shared learnable linear transformation is applied to every node, parameterized by weight vector *W* ∈ *R*^*F*=*F*′^. A self-attention mechanism (*a)* is employed on the nodes, wherein an attention coefficient *e*_*ij*_ is computed which indicates the importance of a neighborhood node j’s features on node i.


(1) $$e_{i\,j}=a\,\left(W\,{\vec{h}}_{i},W\,{\vec{h}}_{j}\right)$$

In the most basic form of the model, every node attends to every node in its neighborhood, where the neighborhood is defined by the first-order neighbors of node i, thereby considering the graph structure into the attention mechanism. In order to make coefficients comparable across different nodes, all choices for j nodes are normalized using a softmax function. The attention mechanism is a single-layer feedforward network, parameterized by vector *a* and containing a leaky rectified linear unit (ReLU) non-linearity. The fully expanded expression of normalized attention coefficients is:


(2) $$a_{\mathrm{ij}}={\mathrm{softmax}}_{j}\,\left(e_{\mathrm{ij}}\right)=\frac{e\,x\,p\,\left(L\,e\,a\,k\,y\,R\,e\,L\,U\,\left({\vec{a}}^{T}\,\left[W\,{\vec{h}}_{i}∥W\,{\vec{h}}_{j}\right]\right)\right)}{\sum_{k\in N_{i}}e\,x\,p\,\left(L\,e\,a\,k\,y\,R\,e\,L\,U\,\left({\vec{a}}^{T}\,\left[W\,{\vec{h}}_{i}∥W\,{\vec{h}}_{k}\right]\right)\right)}$$

Finally, the normalized attention coefficients are used to compute the output features (${\vec{h}}^{\prime }$) for every node after applying a non-linearity.


(3) $${\vec{h}}_{i}^{\prime }=\sigma \,\left(\sum_{j\in N_{i}}\alpha_{i\,j}\,W\,{\vec{h}}_{j}\right)$$

Moreover, to stabilize the attention mechanism, a multihead attention is employed, where p independent attention mechanisms are computed for a node parallelly, each generating a feature vector ${\vec{h}}_{i}^{\prime }$, as shown in Equation (3). The features from each head are concatenated, resulting in the final output with *PF*′ features, as shown in Equation (4):


(4) $${\vec{h}}_{i}^{\prime }=\sigma \,\left(\frac{1}{P}\,\sum_{p=1}^{P}\sum_{j\,\epsilon \,N_{i}}\alpha_{i\,j}^{p}\,W^{p}\,{\vec{h}}_{j}\right)$$

where $\alpha_{i\,j}^{p}$ is the *^pth^* attention mechanism and W*^p^* is the corresponding linear transformation weight matrix. For a node classification task, the GAT model averages multihead attention coefficients from the output layer.

#### Graph Classification Using Graph Attention Network

**FIGURE 1:**
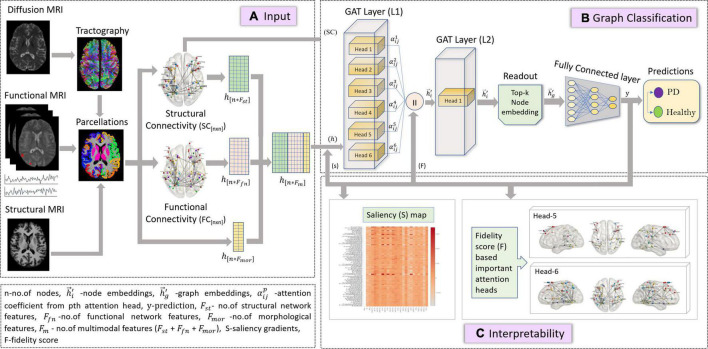
The figure represents proposed the GAT model with multimodal input set along with a graph classification and interpretability framework. Section **(A)** is the input framework that represents a graph matrix and feature set as inputs to the GAT model. Diffusion MRI scans are preprocessed, and tractography is performed using a probabilistic tractography algorithm. Structural connectivity (SC) is computed by employing an atlas brain parcellation from a structural T1-weighted MRI, on the constructed fiber tract to obtain a [n × n] SC matrix with n no. of nodes. Similarly, the preprocessed functional time series from the fMRI scans are used along with the atlas-based brain parcellations to compute an n × n FC matrix. The SC is the input graph matrix provided to the model. The feature inputs (h) to the model contains concatenated SC- and FC-based network features (Fst and Ffn) and morphological features obtained from structural scans for all n nodes. Section **(B)** indicates the GAT-based graph classification framework, which includes the GAT model with an input layer (L1) with 6 attention heads, whose outputs are concatenated and provided as input (h) to the output GAT layer (L2) containing a single head. The output node embeddings from L2 are given to a readout module which employs a top-k mechanism on all node embeddings to extract the top-k most important node embeddings that represent a graph-level embedding (hg). These graph embeddings are then given as input to the fully connected layer which generates output predictions (y) as PD or Healthy. Section **(C)** is the interpretability framework that represents the explainability methods employed on the GAT model to acquire discriminative inputs. The saliency map represents the gradient values that indicate the contribution of each nodal feature toward an accurate prediction; here the *x*-axis represents the features and *y*-axis represents the nodes. The fidelity score generates the most discriminative attention heads or mechanisms that largely contribute toward accurate model predictions.

In this study, we employed the GAT model described in section “Graph Attention Network” for a graph classification task, as shown in Figure 1. The node embeddings from the output GAT layer are aggregated using a top-k readout function to generate graph-level embeddings, which are taken forward for classification. The graph embeddings characterize an individual graph based on its structure and relation between neighboring node features. The input to our proposed GAT model is {*(G (n,e)*), h,*y*}_*s*_ where G_{nxn}_ denotes an undirected graph which is the SC matrix, containing *n* = 86 nodes from the Desikan Killiany atlas, *e* edges indicate the strength of connections between different nodes for s no. of subjects, and $h=\left\{{\vec{h}}_{1,}{\vec{h}}_{2,},..,{\vec{h}}_{n,}\right\}$, *h*_*i*_ ∈ *R^Fm^*, denotes the multimodal feature set (F_*m*_) containing 24 features including morphological (F_*mor*_), structural (F_*st*_), and functional (F_*fn*_) features for n nodes and *y* indicates group labels. The higher-level node features generated from each head in a GAT layer are concatenated and inputted to the next layer. The features at the output layer, ${\vec{h}}_{i}^{\prime }$, from Equation (4) are given to a readout module which extracts the top k no. of nodes for each embedding. These top k node embeddings provide a graph-level representation that encapsulates the most discriminative node features, while lowering the feature dimensionality that may eventually prevent overfitting of the model. The final graph embeddings${\vec{h}}_{g}^{\prime }$ for each graph *g* are obtained by concatenating the top k node embeddings. Subsequently, graph embeddings are passed through a fully connected neural network layer with a softmax activation on the last layer, to generate predictions.

#### Interpretability of the Graph Attention Network Model

Attention mechanism employed by the GAT model is one of its distinguishing attributes over other GNN models, as it can facilitate an in-depth understanding of the model’s working and can highlight the parts of data that the model focuses on while generating predictions. The multihead attention evaluates multiple aspects of the datasets parallelly, capturing different characteristics or complementary attributes within the same dataset. This novel attention mechanism of the GAT model can highlight the most distinguishing brain regions and the interactions between them that contributes to the classification accuracy. However, GAT computes several attention mechanisms at each head and the most relevant attention mechanism can be determined using a fidelity score F.


(5) $$F=\frac{1}{N}\sum_{i=1}^{N}\left(1\left({\hat{y}}_{i}=y_{i}\right)-1\left({\hat{y}}_{i}^{1-m_{i}}=y_{i}\right)\right)$$

where *N* = no. of graphs, *y_i_* is the original accuracy of graph i, *m_i_* is the mask for attention weights, and ${\hat{y}}_{i}^{1-m_{i}}$ is the accuracy obtained when removing the masked attention weights. The fidelity score measures the difference of accuracy between the model’s original predictions and the new predictions obtained by masking each attention head (Pope et al., 2019). The higher fidelity score for a head indicates that the particular attention mechanism employed by the model provides maximum contribution toward classification accuracy. The attention map is a directional plot of attention coefficients for nodes in a graph, consisting of source and destination nodes that indicate the nature of interaction or dependency between nodes. Another interpretability measure employed in this study is saliency analysis which computes the gradients by back-propagating from the model predictions to the multimodal input features of the graph as given by $S=\left(\frac{\partial y}{\partial h}\right)$, where *y* indicates model predictions and *h* indicates set of input features (Pope et al., 2019). Higher gradient values indicate higher contribution of the particular node’s input feature toward an accurate prediction.

### Experiments

Our multimodal model (GAT-SCfs) containing the SC matrix and multimodal feature set was compared against another multimodal model, GAT-FCfs containing the FC matrix and multimodal feature set, to assess the influence of structural and functional neighborhood in classification of PD. To evaluate the classification performance of multimodal feature set over a unimodal feature set, we implemented a purely unimodal structural (GAT-SC) and functional (GAT-FC) model using both features and graphs of the respective modality only. To overcome data imbalance, we performed synthetic minority oversampling technique (SMOTE)-based augmentation on the flattened SC and FC matrices and morphological features, while the remaining network-based nodal features were computed on the augmented graph datasets. A total data of 150 samples (HC-75, PD-75) were used, out of which 10% was kept aside as testing dataset and the remaining data were split into 80% train set and 20% validation set. The feature set was normalized using min–max normalization. Our GAT models contained 2 layers with 6 attention heads in the 1st layer and a single attention head in the output layer, followed by extraction of top 20 (k) node embeddings in the readout module which were given to three fully connected layers for classification. The training details of the model are mentioned in Supplementary Material. The interpretability framework of GAT-SCfs was implemented on the validation dataset. The fidelity score was computed for the 6 attention heads from the first layer, and the top 2 attention heads with the highest fidelity scores were visualized. The average attention maps of the correctly predicted samples were used for visualization. In saliency analysis, we obtained the top most gradients from the average saliency map of correctly predicted samples and correlated them with clinical scores of PD patients using Pearson’s correlation, to evaluate the association between the obtained important features and the progression of PD.

Correlation was performed between moderately high salient node features with a saliency score greater than 0.02 (as seen in Figure 3) and clinical variables such as AAO, DOI, UPDRS-III, H&Y, MMSE, and LEDD score, with a significance threshold of uncorrected *p* < 0.005. Additionally, we compared the classification performance of our proposed multimodal GAT models with other popular graph embedding models such as GCN model, node2vec, and traditional machine learning models like random forest (RF) and multilayer perceptron (MLP). Performance of all models was evaluated using average CV accuracy, test accuracy, and F1 score. The average CV accuracy was calculated by taking the mean of the validation accuracies obtained from all 10 folds, and test accuracy was evaluated on an independent test set. Accuracy is a measure indicating true positive or true negative predictions. The F1 score is defined as the harmonic mean of the model precision and recall and is calculated as F1 score = 2*(Precision*Recall)/(Precision+Recall), where Precision = (TruePositives/TruePositive + FalsePositive) and Recall = (TruePositives/TruePositive+FalseNegative). The F1 score is a better indicator of false positive and false negative predictions. Furthermore, we evaluated the statistical significance for the average CV and test accuracy of the proposed GAT-SCfs model by implementing a non-parametric permutation test, with 1,000 permutations of the labels, which indicates the probability or likelihood of obtaining the accuracy by chance. The input dataset for GCN models was the same as the GAT models, and the node2vec model contained the SC and FC matrices as input to generate multimodal nod embeddings, whereas for traditional machine learnings, the model multimodal feature set comprising both structural and functional network features was used. Details on the training of the comparative models and permutation testing on test accuracy are provided in Supplementary Material.

## Results

**FIGURE 2:**
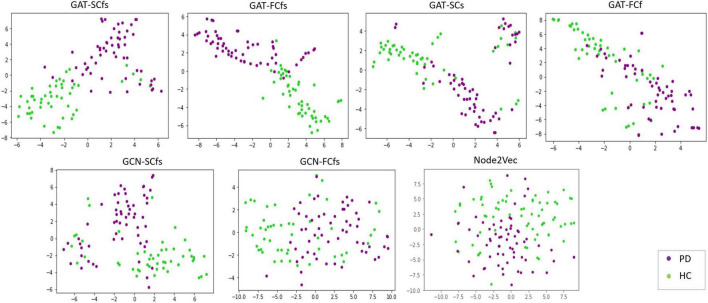
Tsne visualizations of graph embeddings obtained from multimodal and unimodal GAT models as well as from comparative graph-based models such as GCN and node2 Vec.

Detailed demographics and clinical data of PD patients and HC groups involved in this study are shown in Table 1. The classification performance of the multimodal GAT-SCfs model yielded a higher test accuracy of 73% (permutation testing *p*-value = 0.02), 10-fold CV accuracy, and F1 score of 86% (permutation testing *p*-value < 0.001) as compared to the GAT-FCfs multimodal model which showed a CV accuracy of 83% and an F1 score of 81%, as shown in Table 1. The multimodal model GAT-SCfs also provided superior accuracy over its unimodal model, GAT-SC, which gave an accuracy of 79%. Similar performance was shown by FC-based multimodal and unimodal GAT models as shown in Table 2. Thus, both structure and function multimodal models outperformed the unimodal models. Comparison of GAT models with existing state-of-the-art graph embedding models such as GCN and node2vec and traditional machine learning models is also shown in Table 2. Both SC- and FC-based multimodal GAT models performed substantially better than the multimodal GCN models of the respective modalities, on the average CV accuracy. The multimodal node2vec model displayed fluctuating accuracy in the range of 80–90% at every run. Although multimodal node2vec performed marginally better than the proposed multimodal GAT-SCfs model at some instances, the architecture of the model was highly unstable and the construction of random walks is computationally extensive. The t-sne visualization of graph embeddings for all multimodal GAT, GCN, and node2vec models and unimodal GAT models is shown in Figure 2, which further supports the discriminative ability of the GAT-SCfs model with fewer false positives. The GAT-SCfs model showed higher average CV accuracy and F1 score as compared to RF and MLP models. However, the RF and node2vec models yielded a higher test accuracy. Performance of comparative models on the unimodal feature set is provided in Supplementary Material, which illustrates that as compared to GNN architectures, the traditional ML models could provide higher classification accuracy using a unimodal feature, particularly the structural features as compared to when using a multimodal feature set. Overall, our experimental findings demonstrate that the multimodal MRI features of structurally defined neighboring brain regions were able to predict PD pathology more accurately as compared to a unimodal feature set.

**TABLE 2:** Classification results of the proposed GAT model and comparative models.

Sr. no.	Model	CV accuracy	F1-score (CV)	Test accuracy
**Classification performance of GAT models**				
1	GAT-SCfs	86	86	73
2	GAT-FCfs	83	81	60
3	GAT-SC	79	80	60
4	GAT-FC	72	69	66
**Classification performance of comparative models**				
5	GCN-SCfs	76	69	53
6	GCN-FCfs	77	66	66
7	Node2Vec	85	80	80
8	RF	83	83	80
9	MLP	82	84	66

**FIGURE 3:**
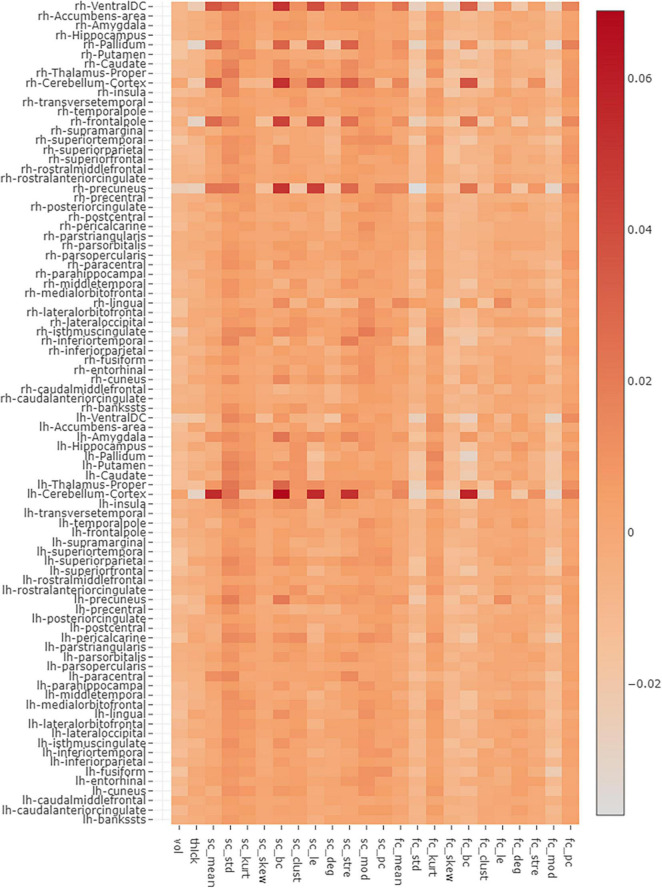
The figure represents an average saliency map of all correctly predicted samples. The map indicates the gradient values, conveying the importance of each nodal feature toward an accurate model prediction. The *x*-axis holds the multimodal features while the *y*-axis indicates the nodes.

**FIGURE 4:**
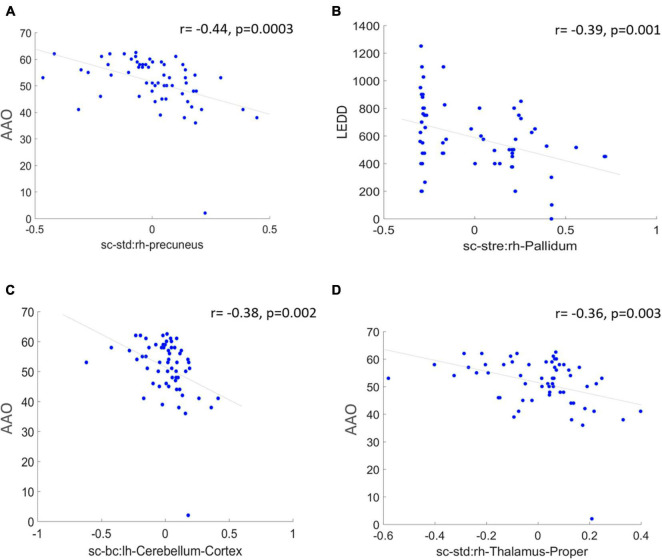
The figure represents correlation between high saliency nodal features and clinical measures at a significance of uncorrected *p* < 0.005. **(A,C,D)** Represent a negative correlation of AAO with nodes such as right hemispheric precuneus, cerebellum, and thalamus proper, and **(B)** indicates a negative correlation between LEDD score and right pallidum.

Saliency maps highlight the most discriminative nodes and features contributing toward the model’s classification performance. The saliency analysis of the GAT-SCfs model shown in Figure 3 indicates that the node-bilateral cerebellum, precuneus, frontal pole, pallidum, and ventral diencephalon played a major role in correctly predicting PD and HC groups. Among the features, SC_mean, SC_S, SC_BC, and FC_BC were the most distinguishing features of the abovementioned brain regions. Occurrence of SC_BC and FC_BC implies that a joint structural and functional influence of these nodes plays a major role in classifying PD. The topmost gradients of the saliency matrix, obtained at a threshold of 0.02, further showed a negative correlation (uncorrected *p* < 0.005) with AAO scores as shown in Figure 4, indicating that the later onset of PD was associated with reduced structural influence of precuneus, cerebellum, and thalamus, and the LEDD score was associated with SC strength of the pallidum.

**FIGURE 5:**
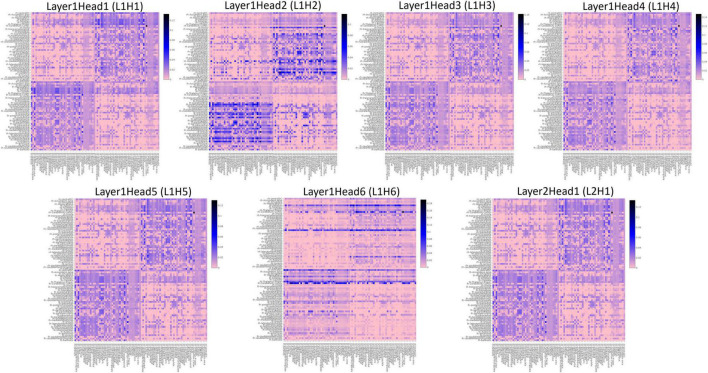
Heatmaps indicating the pairwise attention weights between nodes, generated by each attention head (H1–H6), at every layer (L1, L2) of the proposed GAT model.

**FIGURE 6:**
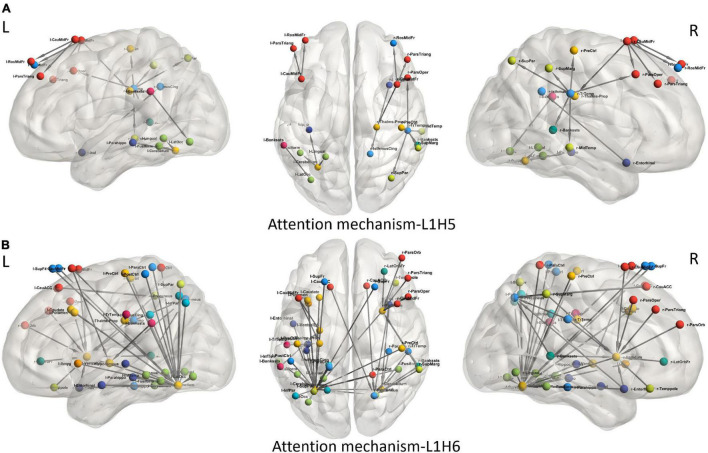
Visualization of attention weights between pairs of brain regions, obtained from top two attention heads (L1H5 and L1H6). **(A)** Attention mechanism L1H5 illustrates the major role of frontal, SMA and temporal regions, while **(B)** Attention mechanism L1H6 illustrates the influence of cerebello-cortical regions in correctly predicting PD.

To understand the different attention mechanisms employed by the GAT-SCfs model, we visualized the averaged attention weight matrix of the correctly predicted data. Attention weights are represented as a symmetric weight matrix of 86 × 86 dimensions, where the edges indicate the bidirectional attention employed by the source node to the destination node. The attention weights from each head at each layer are displayed in Figure 5 using a heatmap, where the *y*-axis denotes source nodes and the *x*-axis denotes destination nodes. Fidelity scores obtained on the heads of the 1st layer were head-1 = 0.14, head-2 = 0.11, head-3 = 0.14, head-4 = 0.11, head-5 = 0.40, and head-6 = 0.40. The attention mechanism of head-5 (L1H5) and head-6 (L1H6) from the 1st GAT layer contributed maximum toward correctly predicting PD and HC groups. The attention mechanism from L1H5 was taken forward by the final output GAT layer as indicated by the similar heatmaps of attention head in the output layer (L2H1) and head-5 (L1H5). Overall, L1H4 shows large dependency between regions of the same hemisphere, particularly between the cerebellum, basal ganglia regions such as pallidum, putamen, caudate, and thalamus proper, and several other cortical regions. L1H6 illustrates a strong influence of specific regions such as basal ganglia regions, precuneus, thalamus, and bilateral cerebellum on widespread areas of the brain mainly comprising the default mode network (DMN) and executive and visual networks. Figure 6 shows the topmost attention weights, thresholded at 0.045 for L1H5 and 0.085 for L1H6 for a clearer visualization and interpretation. These findings are concordant with the existing literature indicating the disruption of corticostriatal circuits in PD.

## Discussion

In this work, we propose the utility of the GNN-based graph classification framework using GAT architecture along with multimodal structure–function brain network features for accurately predicting PD, with a comprehensive interpretability framework. Our proposed model implemented using a structural neighborhood, and multimodal feature sets yielded superior classification performance over a GAT model with a unimodal feature set. The interpretability framework consisting of saliency analysis illustrated the structure–function topological influence of the basal ganglia and cerebello-thalamo-cortical (CTC) regions in delineating PD patients and the relation between their structural alterations and clinical score. Attention maps with high fidelity highlighted the key relations between regions of the motor network, executive network, DMN, and visual network which contributed toward an accurate classification of PD. Our findings of a joint structural and functional involvement of several cortical and subcortical regions demonstrates the utility of a multimodal connectomic framework in predicting PD pathology.

Our proposed GAT-SCfs model demonstrated higher classification performance with a maximum 10-fold CV accuracy 86%, as compared to other comparative models as shown in Table 2 and Figure 3. GAT-SCfs also displayed higher sensitivity and specificity in classifying PD, depicted through its high F1 score. Results suggest that extending the original GAT architecture that was designed for a node classification task, to an end-to-end graph classification task by adding a readout module that generates graph embeddings by aggregating the topmost node embeddings, could become a potential candidate in multimodal brain connectomic analysis. Moreover, the proposed interpretability framework that makes use of attention mechanism on a graph dataset could shed light on the relevant nodal interaction or dependencies of a particular neuropathology. As hypothesized, the GAT-SCfs model with a structurally connected neighborhood showed higher predictive capability, probably indicating that the disrupted structural connections and related functional deficits provide discriminative information on the structure–function relationship, specific to pathology. Both multimodal models, GAT-SCfs and GAT-FCfs, showed a higher accuracy over unimodal models, supporting the utility of multimodal features in characterizing the pathophysiology of PD. The multimodal GAT models showed higher CV accuracy compared to GCN, node2vec, and traditional machine learning models. These findings suggest a superior ability of GAT architecture over GCN in disorders involving multiple network disruptions, which are captured through multiple attention mechanisms. Unlike the GNN models, the node2vec model does not include node features and relies on a random walk-based strategy for optimizing the local neighborhood, to generate node embeddings. The higher test accuracy and moderately high CV accuracy of RF and node2vec models suggest its comparable performance with GAT-SCfs in predicting PD. However, the GAT model employs an attention mechanism on graph, which provides different relations or interactions between nodes, similar to a subnetwork, associated with the pathology. The multimodal node2vec model displayed variable accuracies at every run, probably due to the non-robustness of the random walks algorithm, and thus led to an unstable classification outcome.

Our interpretability framework attributed to the GAT model facilitated detailed understanding of the model predictions. The saliency analysis method back-propagates from model predictions to the input node features, in order to map the influence of each input on the final outcome. The saliency map in Figure 2 illustrated a high structural and functional influence of the cerebellum in predicting PD along with other motor network regions such as the pallidum, ventral diencephalon, and precuneus from DMN and the frontal pole from the executive network. Anatomical and functional studies have suggested that altered basal ganglia and cerebello-thalamo-cortical loops underlie the pathophysiology of motor symptoms such as tremor in PD (Middleton and Strick, 2000; Wu et al., 2012; Lewis et al., 2013; Simioni et al., 2015; Wang et al., 2016; Barbagallo et al., 2017). Abnormal DMN connectivity has also been observed in PD patients (van Eimeren et al., 2009; Yao et al., 2014). The saliency map findings are also in line with existing literature that reports abnormal local network measures such as centrality and local efficiency in structural (Li et al., 2017; Shah et al., 2017) and functional (Tessitore et al., 2019) brain networks of PD patients. A high gradient on the statistical measure SC_mean suggests that the whole-brain SC of the abovementioned regions could be altered in PD. Overall, the saliency map portrayed a higher influence of structural network measures as compared to functional ones in correctly predicting PD and HC groups. A moderately high gradient on FC kurtosis may indicate large variability of functional network measures across subjects. Additionally, on assessing the relationship between the salient nodal features and PD pathology, we found that the structural influence of the precuneus, cerebellum, and thalamus-proper was negatively associated with age of onset of PD, indicating that the structural influence of these regions was lower in patients with a late onset of PD. The LEDD score correlated negatively with SC strength of the pallidum, which may indicate a normalization effect of medication.

We leveraged the multiple attention mechanisms of GAT for a spatial interpretation, which indicates the relation between neighboring brain regions, based on their features. Among the multiple attentions computed by the model, we analyze the two most relevant attention mechanisms (L1H5 and L1H6) that largely contribute toward correct prediction of PD and HC groups. L1H4 showed a distinctly high intrahemispheric relation between brain regions. Figure 6A highlights the topmost attention weights and illustrates the relation between cerebellum and the executive, visual, and DMN regions. This attention mechanism reflects disruption of several brain networks, possibly demonstrating the large-scale network deficits associated with non-motor symptoms in PD. Attention mechanism L1H6 indicates widespread influence of specific brain regions. Figure 6B displays the influential role of the left cerebellum, right pallidum, and precuneus. Specifically, the right pallidum plays an instrumental role on the characteristics of frontal and temporal regions, whereas the cerebellum plays an influential role on multiple brain regions. These findings provide additional support to the key role of the cerebellum and basal ganglia regions that influence or trigger the functionality of supplementary motor areas, DMN, motor, and executive and visual network regions in PD.

This study has a few limitations. Firstly, the absolute thresholding of connectivity matrices may introduce a bias in the input to the GAT model. However, the field of brain connectomics have not yet reached a consensus on the optimal thresholding strategy for functional and structural brain networks. We choose the proportional thresholding technique, with a liberal threshold that approximately equalizes the matrix density of SC and FC at 50%, to retain high as well as moderately strong connections of the matrix. Also, it is reported that high thresholding of the FC matrix, approximately up to 50% density, depicts small world properties in functional brain networks (Achard et al., 2006; Van Den Heuvel et al., 2009; Fornito et al., 2010). Secondly, our acquired functional and diffusion data did not involve high temporal and angular resolution, respectively; therefore, the low quality of functional EPI and DWI acquisitions may limit the robustness of functional correlations and the diffusion model (Mori, 2007; Berman et al., 2013; Tomasi et al., 2015; Wang et al., 2016). However, the proposed GAT model can be employed on a high-resolution dataset that may highlight more intricate structure–function network characteristics of PD. Third, this study has used MRI-based imaging features in the GAT model; however, addition of demographic or clinical variables such as a severity score could enhance the model’s predictive capability, and future studies could explore the efficient incorporation of these variables in a GNN architecture. Finally, our original dataset had a smaller size that may limit the training efficiency of the GNN model. We tried to mitigate this drawback by performing SMOTE-based augmentation of data prior to implementing the model. Future studies could further explore the effect of different thresholding strategies for structural and functional network on the computation of attention coefficient and performance of the GAT model. Incorporating information of individual edges could further enhance the discriminative ability of the GAT model. Moreover, it would be interesting to explore whether a temporal GAT framework could elucidate dynamic network changes in the brain related to network disorders.

## Conclusion

In this study, we proposed a novel GAT-based graph classification model using multimodal brain connectomes and a readout module that extracts topmost node embeddings from a graph to predict PD. Our findings demonstrate the utility of the GNN framework, particularly using an attention-based GAT model with structural neighborhood and a multimodal feature set in classifying PD. Moreover, our detailed interpretability framework consisting of saliency analysis and attention maps and highlights the structure–function influence of the basal ganglia regions, cerebellum, and DMN regions in predicting PD pathology. Our model illustrates the high discriminative power of multimodal brain connectomics and GAT architecture in identifying multisystem network abnormalities for robust classification of neuropathologies.

## Data Availability Statement

The raw data supporting the conclusions of this article may be made available upon request to the authors.

## Ethics Statement

The studies involving human participants were reviewed and approved by the Institute Ethics Committee, NIMHANS. The patients/participants provided their written informed consent to participate in this study.

## Author Contributions

ASa, NV, and ASh: data analysis and processing. SP and AL: data acquisition and clinical evaluation. ASa, MI, JS, and PP: manuscript writing and editing. All authors contributed to the article and approved the submitted version.

## Conflict of Interest

The authors declare that the research was conducted in the absence of any commercial or financial relationships that could be construed as a potential conflict of interest.

## Publisher’s Note

All claims expressed in this article are solely those of the authors and do not necessarily represent those of their affiliated organizations, or those of the publisher, the editors and the reviewers. Any product that may be evaluated in this article, or claim that may be made by its manufacturer, is not guaranteed or endorsed by the publisher.
