# Supplementary material for: Multimodal Brain Connectomics-Based Prediction of Parkinson’s Disease Using Graph Attention Networks
Source: Front Neurosci. 2022 Feb 23;15:741489. doi: 10.3389/fnins.2021.741489 (PMC8904413; doi:10.3389/fnins.2021.741489)
Supplement: Supplementary file 1 [file Data_Sheet_1.docx]

Supplementary Material

# Supplementary Data

Methods:

- 1. Multimodal feature set:

Network based nodal features indicating the local topological attributes of a node such as clustering coefficient (CC), betweenness centrality (BC), degree (D), strength of connectivity (S), local efficiency (LE), modularity (Mod) and participation coefficient (PC) were computed using Brain Connectivity Toolbox [ref]. The detailed description of these graph measures is as follows (rubinov,2010)

Notations: N=set of all nodes in the network, *n*=number of nodes, (*i, j*) = link between nodes *i* and *j,* (*i, j*∈N), *w_ij_* =connection weights, *l^w^* is the sum of all weights in the network,*a_ij_* is the connection status between *i* and *j:a_ij_*= 1 when link (*i, j*) exists (when *i* and *j* are neighbours); *a_ij_*= 0 otherwise (*a_ii_* = 0 for all *i*).

Table-S1: Details of network measures computed from structural and functional connectivity matrices

| **Sr.no.** | **Graph Measure** | **Formula** | **Description** |
| --- | --- | --- | --- |
| 1 | Degree (D) | $D^{i}=\sum_{j\in N} a_{ij}$ | - Degree of a node is the no. of links connected to the node. |
| 2 | Clustering coefficient (CC) | ${CC}^{w}=\frac{1}{N}\sum_{j\in N} \frac{2t_{i}^{w}}{k_{i}(k_{i}-1)}$  where, $t_{i}^{w}$=geometric mean of triangles, *k_i_* = degree | - Clustering coefficient is the average intensity(geometric mean) of all triangles associated with each node. - Clustering coefficient of a node indicates how well its neighboring nodes are connected with each other. |
| 3 | Betweenness centrality (BC) | ${BC}_{i}= \frac{1}{(n-1)(n-2)}\sum_{h,j\epsilon N;h\neq j,i;j\neq i} \frac{\rho_{hj}(i)}{\rho_{hj}}$  where*, ρ_hj =_*number of shortest paths between h and j, *ρ_hj_*(i)=number of shortest paths between h and j that pass-through i. | - Betweenness centrality is the fraction of all shortest paths in the network that contain a given node. - Nodes with high betweenness centrality participate in a large number of shortest paths and are more influential |
| 4 | Local efficiency (LE) | ${LE}_{i}= \frac{1}{2}\sum_{i\epsilon N} \frac{\sum_{j,h\epsilon N,j\neq i} \left( w_{ij}w_{ih}\left[ d_{jh}^{w}(N_{i}) \right]^{-1} \right)^{\frac{1}{3}}}{k_{i}(k_{i}-1)}$  $d_{jh}^{w}$ (*N_i_*) =length of the shortest path between j and h, that contains only neighbours of i | - Local efficiency reflects the information flow and connectedness at a local level, it is related to clustering coefficient. |
| 5 | Modularity (Mod) | ${Mod}_{i}= \frac{1}{l^{w}}\sum_{i,j\epsilon N} \left[ w_{ij}-\frac{k_{i}^{w}k_{j}^{w}}{l^{w}} \right]\delta_{m_{i,}m_{j}}$  where*, m_i_*is the module containing node i, and $\delta_{m_{i,}m_{j}}$= 1 if *m_i_* = *m_j_*, and 0 otherwise. | - Modularity of a node is its no. of connections within its own community over the connections with nodes of other communities. - The modularity is a statistic that quantifies the degree to which the network may be subdivided into clearly delineatedcommunities or sub networks. |
| 6 | Participation coefficient (PC) | ${PC}_{i}=1-\sum_{m\epsilon M} \left( \frac{k_{i}^{w}(m)}{k_{i}^{w}} \right)^{2}$  where *M* = set of modules and $k_{i}^{w}(m)$ is the number of links between *i* and all nodes in module *m* | - Participation coefficient is a measure of diversity of intermodular connections of individual nodes. - Nodes with high participation coefficient indicate higher between module connections whereas nodes with low participation coefficient indicate higher within module connections, known as provincial hubs |
| 7 | Strength (S) | S_i_= $\sum_{j\in N} w_{ij}$ | - Node strength is the sum of weights of links connected to the node. |

- 1. Experimental details:

All GAT models were trained with a learning rate of 0.001 using an Adam optimizer and cross entropy loss function. Hyper parameters for the models were no. of layers (l)=2, no. of heads (h)=6, heads in output layer (lo)=1, hidden dimensions (hd)=8, dropouts (do)=0.2 and k no. of top features with k=20. These hyper parameters were finalized after testing with a combination of parameters (l=1,2,3; h=4,6,8, lo=1,4,6; hd=8,32,64).

The GCN model architecture was similar to the GAT model, comprising 2 layers followed by a topk readout function with top 20 nodes and three fully connected layers for classification. The hyperparameters for GCN were- hidden dimensions=128, learning rate=0.001 using an Adam optimizer and cross entropy loss function. For the multimodal node2vec model, embeddings from SC and FC were obtained individually using the node2vec models, with parameters-p=1,q=0.5,walk length=20,no. of walks=200, hidden dimensions=32. Both SC and FC node embeddings were concatenated and given to a 3-layer ANN classifier, with a learning rate of 0.001, dropout=0.3. Random Forest (RF) model was implemented using 50 decision treesGini impurity criteria, whereas the multilayer perceptron (MLP) was implemented as 2-layer neural network and was trained using hidden dimensions= [16, 8], lr=0.001, Adam optimizer for 1000 epochs. Performance of all models were evaluated using train, test and averaged 10-fold cross validation accuracy and F1 score. The computational complexity of our model is similar to the original GAT model. Our multimodal GAT-SCfs model with 10-fold CV took 38 mins to run on a NVIDIA Tesla V100 GPU of 32 GB, with CUDA version 10.0.

We statistically assessed chance performance of the model by performing permutation testing on the average CV accuracy and test accuracy of the proposed model. The non-parametric permutation testing is a classical statistical method that measures the likelihood of obtaining the reported accuracy of the model by chance [https://link.springer.com/chapter/10.1007/978-3-540-45087-0_28]. Here, the null hypothesis assumes that the relationship between data and labels cannot be learned by the trained classifier reliably, whereas the alternative hypothesis is that the trained model learned and mapped characteristics of data, with a certain probability (p-value) of being at risk of a chance performance. We performed permutation testing by randomly changing labels, over 1000 permutation iterations, which yielded a histogram of accuracy values representing performance from the randomly trained classification models. The statistical p-value of the model was estimated as the proportion of times the permuted test statistics, i.e. the accuracy was found to be greater than or same as the original observed accuracy, across the total no. of permutations. We obtained significance on average CV accuracy (avg CV acc=86, p<0.001) and test accuracy as(test acc=73,p<0.02) of the proposed GAT-SCfs model, indicating that the model did not randomly classify the data and was able to identify the relationship between data and labels.

Results:

Performance of GAT-SCfs model over different hyper parameters is shown in Table-S2. The comparative performance of all unimodal models is shown in Table-S3

# Table-S2: Average CV accuracy of GAT-SCfs model evaluated over a set of hyper parameters.

| Avg CV accuracy | Hidden dimensions | No.of heads (layer1,layer2,layer3) | No.of layer | Topk value |
| --- | --- | --- | --- | --- |
| 70 | 64 | 4,4,6 | 3 | 20 |
| 77 | 8 | 6,1 | 2 | 10 |
| 77 | 32 | 6,4 | 2 | 20 |
| 78 | 8 | 4,4,1 | 3 | 20 |
| 80 | 8 | 6,4 | 2 | 20 |
| 82 | 8 | 6,1 | 2 | 30 |
| 82 | 8 | 4,4,6 | 3 | 20 |
| 82 | 8 | 6,6,1 | 3 | 20 |
| 83 | 8 | 4,1 | 2 | 20 |
| 83 | 8 | 4,4,6 | 3 | 20 |
| 84 | 8 | 8,8 | 2 | 20 |
| 85 | 8 | 6,4 | 2 | 20 |
| 85 | 32 | 4,6 | 2 | 20 |
| 85 | 8 | 6,6,1 | 3 | 20 |
| **86** | **8** | **6,1** | **2** | **20** |

Table-S3: Comparative performance of all unimodal models using SC and FC matrix or features.

| Classification performance of GAT models | | | | |
| --- | --- | --- | --- | --- |
| Sr. no. | **Model** | **CV accuracy** | **F1-score (CV)** | **Test accuracy** |
| 1 | GAT-SC | 79 | 80 | 60 |
| 2 | GAT-FC | 72 | 69 | 66 |
| 3 | GCN-SCs | 80 | 75 | 66 |
| 4 | GCN-FCf | 76 | 78 | 66 |
| 5 | Node2vecSC | 82 | 83 | 80 |
| 6 | Node2vecFC | 76 | 77 | 86 |
| 7 | RF_SC | 86 | 85 | 80 |
| 8 | RF_FC | 80 | 80 | 73 |
| 9 | ANN_SC | 79 | 76 | 80 |
| 10 | ANN_FC | 79 | 78 | 60 |
